# Self-competence in death work among health and social care workers: a region-wide survey in Hong Kong

**DOI:** 10.1186/s12904-018-0317-1

**Published:** 2018-04-20

**Authors:** Johnny T. K. Cheung, Doreen W. H. Au, Wallace C. H. Chan, Jenny H. Y. Chan, Kenway Ng, Jean Woo

**Affiliations:** 1 CUHK Jockey Club Institute of Ageing, The Chinese University of Hong Kong, Shatin, Hong Kong; 2 Department of Social Work, The Chinese University of Hong Kong, Shatin, Hong Kong; 3 Department of Medicine & Therapeutics, The Chinese University of Hong Kong, Shatin, Hong Kong

**Keywords:** Death work, Self-competence, Emotional, Existential, Palliative, Health care worker, Social worker, Hong Kong

## Abstract

**Background:**

According to the Quality of Death Index, Hong Kong is lagging behind many other Western and Asian countries in the category of palliative and healthcare. To ensure the provision of high-quality palliative care, it is important to explore the self-competence of health and social care workers in coping with death work including palliative care. This region-wide study aims to assess the level of self-competence with a validated Self-Competence in Death Work Scale (SC-DWS) and examine its correlates.

**Methods:**

The SC-DWS was administered to a cross-sectional convenience sample of health and social care workers across eight healthcare institutions between January and October 2016. Total scores for the 16-item SC-DWS and its Existential and Emotional subscales were calculated. We then examined sociodemographic variables (e.g., age, profession, place of employment) in relation to the total and subscale scores using multiple linear regression. Coding was conducted on responses to a final open-ended question asking about the personal views of the workers towards their self-competence in death work.

**Result:**

We collected data from 885 health and social care workers. Mean score of the SC-DWS was 60.16 (range: 16 – 80), while its Existential and Emotional subscales scored 37.90 (range: 10 – 50) and 14.46 (range: 4 – 20) respectively. Four categories of personal view towards self-competence in death work including (1) personal resources; (2) existential challenges and coping; (3) emotional challenges and coping; and (4) personal recommendations on improving self-competence were identified. In multivariate analyses, workers aged 50 or above, divorced, working in Hospice A, Rehabilitation Hospital B (where a quality improvement initiative in end-of-life care was implemented) and Acute Hospital B (a Christian institution with strong caring culture) and with personal bereavement experience had significantly higher scores, whereas nurses scored significantly lower than less-educated personal care assistants.

**Conclusion:**

There is still room for improvement in self-competence in death work among health and social care workers, particularly the young, nurses and those working in acute hospitals. Future initiatives should involve identifying barriers in individual healthcare institutions. Training of the provision of palliative care is necessary.

## Background

With growing ageing population, quality of life at the end of life becomes a critical issue due to emerging chronic terminal illnesses in developed regions [1]. Given that over 90% of deaths turned up in hospitals [2], there will be a growing demand for palliative care in Hong Kong. However, according to the 2015 Quality of Death Index [3], Hong Kong is ranked 22 overall and 28 in the category of palliative and healthcare, lagging behind many other Western and Asian countries. In particular services for end-stage non-cancer patients are poorly developed compared with palliative services for cancer patients, even though such patients have a high prevalence of symptoms similar to cancer patients.

Health and social care workers play a vitally important role in improving quality of death among terminally ill patients. A local telephone survey reveals that more than 90% of general public preferred waiting for medical staff to initiate end-of-life care arrangement [4]. The result reflects a strong public expectation of the role of healthcare workers in Hong Kong. Doctors, nurses, personal care workers, social workers and all other clinical staff should learn to work with dying patients and bereaved families [5].

Supportive, therapeutic or remedial work in response to death or issues relevant to death was defined as death work [6], including palliative care, advance care planning discussion and bereavement counselling. Yet, health and social care workers may experience intrinsic challenges during death work. For instance, some doctors tended to avoid end-of-life care discussion [7] and regard death work as an act of ‘giving up’ [8]. They were likely to be overwhelmed by emotions and suffering of patients and have their life-and-death assumptions and meaning in life shattered. These findings imply that ‘self-competence’ is crucial for coping with the challenges faced by the workers [6] and ensuring the provision of high-quality death work [9]. In contrast, failure to develop self-competence in death work may lead to burnout and compassion fatigue [10, 11]. Indeed adequate staff training and experience have been identified as one of the key features of quality end-of-life care, and at the same time, the predominant curative focus in medical care forms a barrier [12]. A case note audit of 61 patients with a mean age of 84 years characterized by multi-morbidity and disabilities (54% being residents of long-term care homes) who died in an acute general hospital during the last 180 days, shows that end-of-life care conversations occurred in only 21%, with no patients having an advance care plan or advance directives, even though 79% had a ‘do not resuscitate’ order [13]. There are few measures of competence in caring for patients who are at the end of life.

A group of local researchers in Hong Kong developed and validated a Self-Competence in Death Work Scale (SC-DWS) to assess health and social care workers’ level of self-competence in death work and reflect their needs in facing death [8]. The scale quantifies two key coping resources: (1) existential coping (e.g. answering questions regarding meaning in life and suffering); (2) emotional coping (e.g. relieving intense distress and grief) [6, 14]. Though local studies of SC-DWS have been previously conducted [5, 8], sample sizes of the studies are relatively small and the majority of the study subjects were social workers.

Therefore, the present study aimed to (1) conduct a region-wide survey assessing the self-competence level among various health and social care workers in Hong Kong by the SC-DWS and to (2) examine sociodemographic variables associated with the self-competence level.

## Methods

### Study design and participants

This cross-sectional survey assessed baseline self-competence level in participants of an End-of- life Care Capacity Building Programme, as the first step of a wider quality improvement initiative. The Programme consisted of workshops, seminars, talks, and academic meetings. Participants included healthcare professionals (doctors, nurses and allied health professionals), personal care workers (patient care assistants and healthcare assistants), social workers and other care workers in three acute hospitals, two rehabilitation hospitals, a nursing home, a hospice as well as a Residential Care Home for the Elderly located in New Territories East region of Hong Kong. Data were collected from the participants through convenience sampling between January and October 2016.

### Measurement

The survey adopted the validated SC-DWS consisting of 16 items to assess the self-competence level of death work [8]. Existential subscale (10 items) and Emotional subscale (4 items) of SC-DWS specifically indicate the existential and emotional coping with death work respectively. Internal consistency (Cronbach alpha of 0.88 for entire SC-DWS, 0.84 for Existential subscale, and 0.78 for Emotional subscale), item-total correlation (mean of 0.61, 0.64, and 0.78), construct validity (significant correlations of 0.20 – 0.49, 0.19 – 0.46, and 0.18 – 0.50 with four related scales), and discriminative validity (significant correlations of 0.23, 0.23, and 0.20 with work experience in current occupation and correlations of 0.30, 0.31, and 0.17 with experience in death work) were demonstrated [8].

The questionnaire firstly described death work as “death-related support work done by helping professional, such as palliative care or emotional support”. After that, we asked participants to rate compatibility between their own current attitudes and situations in real life for each item, on a scale of 1 (completely incompatible) to 5 (completely compatible). In addition to quantitative measures, an open-ended question asked the participants to share personal views towards their self-competence. We also collected sociodemographic characteristics of participants including age, gender, marital status, education level, occupation, work experience in current occupation, experience in death work, as well as personal bereavement experience.

### Data analysis

Mean and standard deviation (SD) of total scores of SC-DWS (range: 16 – 80), its Existential subscale (range: 10 – 50), Emotional subscale (range: 4 – 20) were computed by summing corresponding items. A greater score indicated a greater self-competence in death work.

To complement the quantitative statistics above, we analyzed participants’ personal views towards their self-competence level. On the one hand, two authors (J T.K. C and J H.Y. C) deductively coded the majority of the views into categories and subcategories conceptualizing SC-DWS [6, 8]. On the other hand, the authors they inductively coded a few responses regarding participants’ personal recommendations on improving quality of death work.

In addition, we used multiple linear regression and reported unstandardized beta coefficients (B) and standard errors (SE) to identify sociodemographic covariates significantly associated with the mean scores of the SC-DWS and its subscales. Dummy variables of ordinal and nominal covariates containing more than 2 groups (age, marital status, occupation, education, institutions) were created for the regression tests. All statistical analyses were performed by IBM SPSS Statistics 24. A *p*-values < 0.05 and a Cronbach’s α > 0.7 were considered to be statistically significant and internally consistent.

## Results

Table 1 lists characteristics of our participants. Of the 885 participants, the majority were female (81.6%), aged 40 – 49 (29.4%), married (57.1%), nurse (65.3%), degree holders (30.0%), working in Residential Care Home for the Elderly (21.9%), with personal bereavement experience (86.7%) and had more than 10 years of work experience (53.1%) but less than 10 years of death work experience (62.7%).

**Table 1:** Characteristics of participants (*n* = 885)

Variable	N (%)
Gender	
Male	162 (18.4)
Female	720 (81.6)
Age	
18–29	227 (26.1)
30–39	160 (18.4)
40–49	256 (29.4)
≥ 50	228 (26.2)
Marital Status	
Single	341 (39.4)
Married	494 (57.1)
Divorced	30 (3.5)
Occupation	
Doctor	55 (6.3)
Nurse	572 (65.3)
Allied health professional	41 (4.7)
Personal care assistant	149 (17.0)
Social worker and other care worker	59 (6.7)
Education	
Secondary education or below	208 (24.3)
Higher diploma/ associate degree/ certificate course	167 (19.5)
Degree	257 (30.0)
Postgraduate degree	224 (26.2)
Work Experience	
≤ 10 years	380 (46.9)
> 10 years	431 (53.1)
Death Work Experience	
≤ 10 years	405 (62.7)
> 10 years	241 (37.3)
Hospital	
Acute Hospital A	56 (6.3)
Acute Hospital B	115 (13.0)
Acute Hospital C	126 (14.2)
Rehabilitation Hospital A	66 (7.5)
Rehabilitation Hospital B	191 (21.6)
Hospice A	31 (3.5)
Nursing Home A	106 (12.0)
Residential Care Home for the Elderly	194 (21.9)
Personal bereavement experience	
Yes	756 (86.7)
No	116 (13.3)

Table 2 shows descriptive statistics of SC-DWS, its existential subscale, emotional subscale and each item. Mean score of the entire SC-DWS was 60.16 (SD = 8.39), while the scores of the existential and the emotional subscales were 37.90 (SD = 5.33) and 14.46 (SD = 2.59) respectively. Among 16 items, ‘I can fully accept that I cannot completely control life, for example, the life and death of patient/service user’ (item 1) showed the highest mean (4.11) while ‘I have finished most of my business, which has reduced my life regrets’ (item 4) showed the lowest mean (2.91). The entire SC-DWS and the two subscales were all internally consistent (Cronbach’s alpha: 0.90 for entire scale, 0.87 for Existential subscale, and 0.79 for Emotional subscale).

**Table 2:** Descriptive statistics of SC-DWS, its Existential subscale, Emotional subscale and each item

Scale/ Subscales/ Items	Mean (SD)
SC-DWS (Item 1 - 16)	60.16 (8.39)
Existential Subscale (Item 1 - 7, 11-12, 16)	37.90 (5.33)
Emotional Subscale (Item 8 - 10, 14)	14.46 (2.59)
1. I can fully accept that I cannot completely control life, for example, the life and death of patient/service user.	4.11 (0.81)
2. I can fully accept that suffering is inevitable, for example, the suffering of patient/service user during the dying process.	4.06 (0.80)
3. I am prepared for my death and that of my family, for example, being open to discuss death with my family, or think about my funeral.	3.37 (0.99)
4. I have finished most of my business, which has reduced my life regrets.	2.91 (1.06)
5. Confronted by uncertainties in life, I take things less for granted and apply this to my life.	4.05 (0.76)
6. Confronted by uncertainties in life, I live my life more positively and have found my meaning in life.	3.91 (0.76)
7. I can fully accept that my emotions are aroused during work.	3.64 (0.86)
8. I can effectively cope with my emotions induced by work.	3.67 (0.75)
9. I have coped with my bereavement experience or experience related to death.	3.62 (0.93)
10. When I feel stressed by work, I can take care of my needs properly.	3.61 (0.78)
11. I can fully accept the nature of death work, including pity or depressed feelings.	3.77 (0.76)
12. Even though death is inevitable, I admire my contribution to work.	4.08 (0.69)
13. When I feel stressed by work, I can find meaning in work.	3.85 (0.75)
14. I do not bring work-induced emotions into life and do not bring life-induced emotions into work.	3.55 (0.86)
15. I admit that helping professionals are challenged by life and death like ordinary people, so I can accept my limitations in work.	3.96 (0.73)
16. I can fully accept that, even though I am a helping professional, I feel helpless about life and death.	3.99 (0.80)

Table 3 summarizes personal views towards self-competence in death work among health and social care workers. Four categories and 20 subcategories relating to self-competence in death work among the health and social care workers were identified. The categories include (1) personal resources; (2) existential challenges and coping; (3) emotional challenges and coping; and (4) personal recommendations on improving self-competence and quality of death work.

**Table 3:** Personal views towards self-competence in death work among health and social care workers (*n* = 145)

Categories	Subcategories	N (%)
(1) Personal resources	Feeling helpless and perceiving death work as challenging	15 (10.34)
	Compassion	15 (10.34)
	Calmness and maturity	10 (6.90)
	Positive orientation	5 (3.45)
	Willingness to learn	2 (1.38)
	Grief experience	2 (1.38)
(2) Existential challenges and coping	Accepting limits of human existence	30 (20.69)
	Sense of meaningfulness in life	17 (11.72)
	Finding meaning and experiencing passion for their work	13 (8.97)
	Equality in relationship with patients	9 (6.21)
	Accepting inevitability of death	7 (4.83)
	Role of religion	4 (2.76)
(3) Emotional challenges and coping	Inability of detaching themselves from emotions of clients after work	6 (4.14)
	Inability of handling previous bereavement experience	2 (1.38)
	Self-care and maintaining good mental health	1 (0.69)
(4) Personal recommendation	Administrative support/ resource allocation	2 (1.38)
	Emotional regulation training and communication skill training	2 (1.38)
	Cultural change	1 (0.69)
	Euthanasia	1 (0.69)
	Promoting patient-centered care and advance care planning	1 (0.69)

Table 4 presents the results of multiple linear regression of SC-DWS, Existential and Emotional subscale on sociodemographic variables. For the entire SC-DWS and the Existential subscale, participants aged 50 or above, divorced, working in the Acute Hospital B, the Rehabilitation Hospital B, and with personal bereavement experience had significantly higher scores. Meanwhile, nurses scored lower compared to personal care assistants. For the Emotional subscale, participants aged 50 or above, divorced and with personal bereavement experience had significantly higher scores. By contrast, female, nurses, social workers and other care workers had lower scores.

**Table 4:** Multiple linear regression of SC-DWS, Existential and Emotional subscale on sociodemographic variables

Variables	SC-DWS	Existential subscale	Emotional subscale
	B (SE)	B (SE)	B (SE)
Gender			
Male	Referent	Referent	Referent
Female	−0.60 (0.84)	0.05 (0.54)	−0.60 (0.26)*
Age			
18–29	Referent	Referent	Referent
30–39	−0.04 (1.19)	0.29 (0.77)	−0.19 (0.37)
40–49	0.95 (1.47)	1.04 (0.95)	0.06 (0.46)
≥ 50	4.94 (1.57)**	3.20 (1.02)**	1.12 (0.49)*
Marital status			
Single	Referent	Referent	Referent
Married	0.30 (0.83)	−0.07 (0.54)	0.31 (0.26)
Divorced	5.99 (2.13)**	3.84 (1.34)**	1.30 (0.65)*
Occupation			
Personal care assistant	Referent	Referent	Referent
Doctor	−2.17 (1.85)	−0.90 (1.20)	−1.04 (0.58)
Nurse	−4.52 (1.41)***	−2.60 (0.91)**	−1.31 (0.44)**
Allied health professional	−2.19 (2.00)	−1.06 (1.29)	−0.93 (0.63)
Social worker and other care workers	−3.35 (1.77)	−1.87 (1.14)	−1.25 (0.55)*
Education			
Secondary education or below	Referent	Referent	Referent
Higher diploma/ associate degree	−1.47 (1.17)	−0.69 (0.76)	−0.42 (0.36)
Degree	0.13 (1.18)	0.24 (0.76)	−0.34 (0.37)
Postgraduate degree	1.50 (1.22)	0.87 (0.78)	0.22 (0.38)
Work experience			
≤ 10 years	Referent	Referent	Referent
> 10 years	0.23 (1.27)	0.41 (0.82)	−0.15 (0.40)
Death work experience			
≤ 10 years	Referent	Referent	Referent
> 10 years	0.21 (0.92)	0.06 (0.59)	0.16 (0.29)
Institution			
Acute Hospital A	Referent	Referent	Referent
Acute Hospital B	3.63 (1.52)*	2.27 (0.98)*	0.70 (0.48)
Acute Hospital C	2.11 (1.54)	1.42 (1.00)	0.39 (0.48)
Rehabilitation Hospital A	2.09 (1.78)	1.74 (1.16)	0.07 (0.56)
Rehabilitation Hospital B	3.69 (1.47)*	2.11 (0.96)*	0.83 (0.46)
Hospice A	5.50 (1.92)**	3.01 (1.24)*	1.67 (0.60)**
Nursing Home A	−0.11 (1.68)	−0.29 (1.09)	0.00 (0.53)
Residential Care Home for the Elderly	2.40 (1.68)	1.49 (1.09)	0.41 (0.52)
Personal bereavement experience			
No	Referent	Referent	Referent
Yes	2.35 (0.91)**	1.17 (0.59)*	0.78 (0.29)**

*p < 0.05; **p < 0.01, ***p < 0.001

## Discussion

### Self-competence in death work among health and social care workers

The present study is the first region-wide survey assessing the self-competence in death work among health and social care professionals across hospital and community. Comparing with the preliminary study with social workers constituting the majority of the sample [8], our samples with healthcare workers as the majority scored similarly on the SC-DWS (present study: 60.16 vs preliminary study: 60.38), the Existential (37.90 vs 38.32) and the Emotional subscale (14.46 vs 14.05) (Table 2). In view of a larger sample size and a variety of health and social care workers involved, our baseline result can provide a reference point for future studies.

Our study provides further insights into their self-competence level by collecting their personal views towards it (Table 3). Regarding personal resources, a number of participants felt helpless and perceived death work as a challenging duty that they could hardly take control of. On the other hand, positive personal resources including compassion, calmness, maturity and positive orientation were identified from other participants. Regarding existential coping, an acceptance of limits of human existence and a sense of meaningfulness in life were prevalent among the workers. They tended to emphasize quality of life rather than prolonging life. Regarding emotional coping, inability of detaching themselves from emotions of dying patients and bereaved families was a major emotional challenge. This phenomenon was particularly common among healthcare workers providing palliative care [15]. A few of our participants thus call for more training on emotional management, which might reduce burnout among the workers [16].

### Sociodemographic determinants of the self-competence in death work

Age and previous bereavement experience were positively correlated with self-competence in death work (Table 4), consistent with previous studies [5]. Interestingly, work experience and death work experience, which were found to be significant predictors of self-competence in the previous studies [5, 17], were no longer significant in this study. This phenomenon may suggest that personal resources and coping skills were acquired through personal experience especially prior bereavement experience of family members, relatives or friends, which increases with age. On the other hand, health and social care workers who did not have a firsthand experience of bereavement may have lower self-competence. Therefore, apart from emotional management and communication skill suggested by the participants, training provided should also include role-playing exercises putting them into real-life situations (e.g. bereavement) and allowing them to become aware their personal needs.

More interestingly, scores among nurses were significantly lower than personal care assistants in all three SC-DWS and its subscales. The result contradicts the assumption that professionals who received more education would experience a higher level of self-competence in death work. Insufficient training for nurses can be one but not the only reason [18]. The nature of work may also account for the difference. Nurses are responsible for coordinating patient care [19], facilitating end-of-life care discussion and supporting the patients and families [20], while personal care workers assist nurses and doctors and perform other indirect caring duties such as equipment cleaning. Hence, nurses who were more familiar with the medical conditions of and had more contact with terminally ill patients were more likely to be affected by death anxiety and distress [21]. This may contribute to discomfort with communication with patients and their family members about death [22]. Apart from nurses, doctors also had a lower score than personal care assistants although the statistical significance was not reached, perhaps due to the small sample size. These results may indicate an inadequacy of death work-related training in undergraduate medical and nursing curriculum previously suggested [2].

The present study also demonstrates variation across the healthcare institutions in the New Territories East region. Those working in Hospice A, Rehabilitation Hospital B, Acute Hospital B had significantly higher scores in SC-DWS. The results probably reflect difference in overall management policies of end-of-life care. Hospice A is a 25-bed hospice dedicated to caring for the terminally ill. Rehabilitation Hospital B has implemented a continuous quality improvement initiative in improving end-of-life care covering all medical wards for 7 years [23]. Acute Hospital B is run by a Christian organization which had established a very strong caring culture. In contrast, Acute Hospital A, where the workers score relatively low in SC-DWS, are busy with providing care in all specialities with rapid turnover, and little focus has been placed on end-of-life care. The variation in SC-DWS scores may suggest that provision of end-of-life care by individual institution needs to be part of quality improvement initiatives, with staff training supported by changes in service provision systems such as documentation of serious illness conversations, advance care planning and advance directives. Such improvement initiatives may vary among different institutions since they have different service demands and staff culture. The latter factor needs to be taken into account in any strategies for improvement in staff competence.

### Limitation and further study

Our study has several limitations. First, the participants might have given desirable answers, attributable to the self-reporting nature and a perceived role as ‘competent professionals’ who should be well-prepared for death work. Second, participants of the end-of-life workshops, seminars, talks and academic meetings might have been more positive towards or familiar with death work. Both the social desirability and the participation bias could contribute to an overestimation of the SC-DWS score. Third, convenience sampling might diminish the representativeness of our sample. For example, there was a lack of participation among doctors, for reasons that were unclear. Fourth, the response rate of the open-ended question collecting personal views towards death work was relatively low, possibly due to lack of interest in, familiarity with, or strong views towards this topic.

Future quantitative studies can assess SC-DWS in other sociocultural contexts and in more representative samples. Qualitative studies should be conducted to explore factors underlying the self-competence level and examine whether their experience in death work is consistent with our findings.

## Conclusion

The present study assesses self-competence in death work among health and social care workers in the North Territories East region of Hong Kong. Our results show that there is room for improvement in the self-competence level among the workers, especially the young, nurses and those working in acute hospitals. Future studies can assess SC-DWS in other sociocultural contexts and explore factors underlying the self-competence level. Future initiatives should involve identifying barriers in individual healthcare institutions and training of the provision of palliative care.

## Abbreviations

B: Unstandardized beta coefficient.
SC-DWS: Self-competence competence in death work scale
SD: Standard deviation
SE: Standard error
